# Mercury, Food Webs, and Marine Mammals: Implications of Diet and Climate Change for Human Health

**DOI:** 10.1289/ehp.7603

**Published:** 2005-02-02

**Authors:** Shawn Booth, Dirk Zeller

**Affiliations:** Fisheries Centre, University of British Columbia, Vancouver, British Columbia, Canada

**Keywords:** climate change, Ecopath, Ecosim, Ecotracer, mercury, pollutant, trophic modeling

## Abstract

We modeled the flow of methyl mercury, a toxic global pollutant, in the Faroe Islands marine ecosystem and compared average human methyl mercury exposure from consumption of pilot whale meat and fish (cod, *Gadus morhua*) with current tolerable weekly intake (TWI) levels. Under present conditions and climate change scenarios, methyl mercury increased in the ecosystem, translating into increased human exposure over time. However, we saw greater changes as a result of changing fishing mortalities. A large portion of the general human population exceed the TWI levels set by the World Health Organization [WHO; 1.6 μg/kg body weight (bw)], and they all exceed the reference dose (RfD) of 0.1 μg/kg bw/day set by the U.S. Environmental Protection Agency (EPA; equivalent to a TWI of 0.7 μg/kg bw). As a result of an independent study documenting that Faroese children exposed prenatally to methyl mercury had reduced cognitive abilities, pregnant women have decreased their intake of whale meat and were below the TWI levels set by the WHO and the U.S. EPA. Cod had approximately 95% lower methyl mercury concentrations than did pilot whale. Thus, the high and harmful levels of methyl mercury in the diet of Faroe Islanders are driven by whale meat consumption, and the increasing impact of climate change is likely to exacerbate this situation. Significantly, base inflow rates of mercury into the environment would need to be reduced by approximately 50% to ensure levels of intake below the WHO TWI levels, given current levels of whale consumption.

Although occurring naturally [United Nations Environment Programme (UNEP) 2002], mercury is a global pollutant and concerns public health when it is elevated above natural background levels, mainly through anthropogenic causes (Boening 2000). The cycling of mercury through the marine environment involves different chemical forms (Morel et al. 1998). In marine organisms, it is most commonly found as mono-methyl mercury (CH_3_Hg^+^) or as mercury ion (Hg^2+^; Downs et al. 1998; Morel et al. 1998). Generally, it is monomethyl mercury that is of concern because it bioaccumulates and biomagnifies at all trophic levels in the food web and can have severe toxicologic effects. Methyl mercury first gained notoriety in Minimata, Japan, after causing severe disabilities and death among people eating seafood contaminated through industrial mercury discharge accumulating through the food chain (Fujuki 1980).

Mercury concentrates in the marine environment, especially in deep ocean waters, which contain approximately 74% of the global total, compared with approximately 24 and 2% in the shallow part of the oceans and the atmosphere, respectively (Mason and Sheu 2002; Morel et al. 1998). A large portion of mercury in the ocean is transformed to Hg^2+^ and becomes available for methylation (Fitzgerald and Mason 1997). Thus, methyl mercury concentrations are primarily a function of methylation and demethylation rates (Morel et al. 1998) and of sedimentation and food chain uptake (Fitzgerald and Mason 1997).

Methylation seems driven by biotic processes (UNEP 2002) and has been linked to sediment-bound sulfate-reducing bacteria (King et al. 2001). However, methylation is also thought to occur throughout the water column (Morel et al. 1998). Significant in light of global climate change, methylation rates are temperature dependent (Downs et al. 1998). Concentrations of mercury measured in the North Atlantic Ocean averaged approximately 1 pM (Mason et al. 1998; Mason R, personal communication), and usually 80–99% of mercury found in fish muscle tissue is methyl mercury, regardless of its concentration in the environment (Downs et al. 1998).

The population of the Faroe Islands (northeast Atlantic, 62°N, 7°W) relies heavily on marine resources, both for consumption and as a key economic activity. Fisheries account for more than 95% of exports and 44.5% of gross domestic product, with demersal species (e.g., Atlantic cod, *Gadus morhua*) the most important grouping (Zeller and Reinert 2004). There is a long tradition of hunting pilot whales (*Globicephala melas*), with records back to 1709 (Faroe Government 2004). Today, whale meat accounts for approximately 30% of total meat produced on the islands (Faroe Government 2004) and is a cultural component of the Faroe lifestyle. It is made available using a free, traditional distribution system (Bloch and Zachariassen 1989).

In the 1990s, Grandjean et al. (1992, 1997) documented cognitive impairment in a cohort of Faroese children who were exposed to elevated levels of methyl mercury prenatally, based on consumption of whale meat during pregnancy. Subsequent studies also provided evidence of attenuated postnatal growth of breast-fed children due to contaminant loading of human milk via maternal seafood diet (Grandjean et al. 2003). The average daily seafood consumption by adults was reported as 12 g whale muscle and 72 g fish (Vestergaard and Zachariassen 1987), with the majority of fish consumed being cod (Grandjean et al. 1992). In response to Grandjean et al. (1992), the diet of pregnant women has since changed to an average daily consumption of 1.45 g whale muscle and 40.2 g fish (Weihe et al. 2003), with an associated decrease in mercury assimilation (Weihe et al. 2005).

We modeled the transfer of methyl mercury through the food web in the marine ecosystem of the Faroe Islands using Ecotracer, a novel routine of the trophic ecosystem modeling approach Ecopath with Ecosim (Christensen and Walters 2004). A published Ecopath model for the Faroe Islands marine ecosystem (Zeller and Reinert 2004) was slightly modified to 21 functional groups plus detritus, with a functional group consisting of either a single species (e.g., cod, *Gadus morhua*) or a group of species (e.g., plankton). We initiated model simulations with a 100-year baseline run (*t*_0_ – *t*_100_) using only estimated mercury base inflow rate changes and environmental concentrations based on Mason and Sheu (2002). This baseline simulated a bottom-up approach to reach species/functional group methyl mercury concentrations comparable with field observations. We then performed impact simulations (*t*_100_ – *t*_200_) to evaluate changes in fishing mortality rates and effects of increased sea temperatures due to climate change on methyl mercury bioaccumulation in all species/groups. We evaluated results in the context of human dietary consumption compared with standardized tolerable weekly intake (TWI) limits, based on the World Health Organization (WHO) equivalent [1.6 μg/kg body weight (bw); Food and Agriculture Organization of the United Nations (FAO)/WHO 2003] and the U.S. Environmental Protection Agency (EPA) equivalent [0.7 μg/kg bw converted from the reference dose (RfD) of 0.1 μg/kg bw/day; Electric Power Research Institute (EPRI) 2004]. Furthermore, we derived a functional relationship to predict methyl mercury concentrations in fish species based on growth and life history parameters such as trophic level (TL), consumption to production ratio (*Q*/*P*), and the von Bertalanffy growth coefficient (*K*).

## Materials and Methods

### Modeling approach.

Using Ecotracer (Christensen and Walters 2004), we traced the transfer and bioaccumulation of methyl mercury through all ecosystem components (functional groups, composed of either individual species or species groups) based on diet transfers and direct uptake from the environment. Underlying Ecotracer was a trophic marine ecosystem model modified from Zeller and Reinert (2004; see Supplemental Material http://ehp.niehs.nih.gov/docs/2005/7603/suppl.pdf).

Within Ecotracer, the concentration of a contaminant in a given species or group of species is expressed as a function of gains from direct uptake from the environment and from the uptake from each food item as defined in the ecosystem model’s diet matrix, versus losses due to instantaneous decay rates, unassimilated food, predation, and instantaneous nonpredation death rates (Christensen and Walters 2004). Four contaminant parameters must be provided: *a*) initial concentrations for each species/group, including environmental concentrations; *b*) direct uptake rate parameters for each species/group; *c*) concentrations per biomass in immigrating organisms; and *d*) metabolism/decay rates for each species/group. Unassimilated food and instantaneous nonpredation death rates are estimated by the modeling routine.

### Ecotracer input data.

#### Base inflow rate.

The Ecotracer input data derived as described below are presented in Table 1. The base inflow rate is the sum of the methylation rate occurring in the sediments, the demethylation rate by chemical transformation at the sediment–water interface, and the net methylation rate occurring in the water column. Approximately 2% of the total mercury flux is being methylated per year (Fitzgerald and Mason 1997). The present day wet depositional flux is estimated to be approximately 7.7 × 10^−6^ g/m^2^ for latitudes 30° to 70° N, and approximately 0.68 × 10^−6^ g/m^2^ for latitudes 70° to 90° N (Downs et al. 1998). Given that the Faroe Islands are located at 62° N, we assumed that the base inflow rate would be intermediate to these two values. A base inflow rate of 0.113 g/km^2^/year, equivalent to a wet depositional flux of 5.6 × 10^−6^ g/m^2^, best accounted for empirically measured environmental concentrations and compared favorably with our assumption of an intermediate value. Dry depositions were not considered because they are not deemed to be important in oceans (Mason et al. 1994).

#### Starting concentrations.

The depth-averaged environmental concentration in the entire global ocean increased by approximately 9% between preindustrial periods and the modern, industrial era (Mason and Sheu 2002), whereas concentrations in near-surface waters are thought to have increased 2- to 3-fold (Mason et al. 1994). The modern environmental concentration by volume was based on current measurements of total mercury for the North Atlantic (~ 1 pM; Mason et al. 1998; Mason R, personal communication), resulting in a methyl mercury volume concentration of 0.02 pM (i.e., 2% of total mercury flux being methylated; Fitzgerald and Mason 1997). Using an average water depth of 838 m [National Oceanic and Atmospheric Administration (NOAA) 2001] and a surface area of approximately 190,200 km^2^ for the model area (Zeller and Reinert 2004), we converted the environmental volume concentration into an environmental area concentration of 3.612 g/km^2^ for the modern, industrialized period. However, to account for increasing baseline flow rates due to industrialization, we set the initial, pre-industrial environmental concentration (*t*_0_) of methyl mercury to 3.312 g/km^2^ (i.e., a value 9% less than the current measurements, representing the 9% increase between pre-industrialized and industrialized periods; Mason and Sheu 2002). Initial species/group concentrations for biota at time *t*_0_ were set at estimated preindustrial period values (Table 1) determined through a prebaseline simulation. In this simulation, the environmental and biota concentrations were initiated at zero and run to the preindustrialized period environmental concentration of 3.312 g/km^2^, while allowing the biological groups to equilibrate, resulting in preindustrialized period estimates for biota.

#### Direct uptake and demethylation rates.

We applied direct uptake rates to phytoplankton, zooplankton, and benthos (Table 1) because this is the dominant entry pathway for mercury accumulation (Canli and Furness 1995; Mason et al. 1996). For higher species and groups, diet accounts for approximately 90% of mercury accumulation (Downs et al. 1998). Therefore, we ignored uptake rates due to respiration.

We used demethylation (decay) rates for marine mammals (Table 1) because they are known to demethylate methyl mercury by forming a mercury selenide complex (Wagemann et al. 1998), although actual rates of demethylation have not been measured in marine mammals. However, preliminary simulations without demethylation indicated that the methyl mercury concentrations in marine mammals increased sharply without reaching a limit. Furthermore, although other species may also have demethylation capabilities, no information in this regard is available.

### Baseline simulations.

We followed the bottom-up flow of methyl mercury through ecosystem components (species/groups) with a 100-year baseline simulation (*t*_0_ – *t*_100_) and compared end concentrations with published field measurements. Transformation of literature values of mercury to methyl mercury were based on Dietz et al. (1996), Andersen and Depledge (1997), and Joiris et al. (1997b).

### Impact simulations.

Building on the *t*_100_ baseline, we simulated the effects of changes in fishing mortality rate (*F* ) on the accumulation of methyl mercury in commercial fish species and pilot whales over a second 100-year period (t_100_ – t_200_). Changing F alters the production to biomass (*P*/*B*) ratio of a species/group (and hence mercury accumulation), because *P*/*B* can be defined as the total mortality rate, which is fishing mortality plus natural mortality (Christensen et al. 2004).

We also investigated how methyl mercury concentrations would change with increased seawater temperatures based on global climate-change scenarios. Increases in temperatures would lead to increases in the methylation rate via the Q_10_ rule, making increased amounts of methyl mercury available for uptake (Downs et al. 1998). The Q_10_ rule relates changes in temperature to changes in the metabolic rates of organisms, whereby a 10°C temperature increase leads to a doubling of the metabolic rate (Randall et al. 2002). Studies on the North Atlantic generally project warming of 0.4–1.0°C per century (International Panel on Climate Change 2001).

#### Human dietary intake.

We modeled the dietary intake (DI) of methyl mercury as


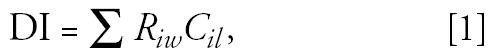


where *R_iw_* is the daily intake (in grams) of seafood in weight *w* of species *i*, and *C_il_* is the concentration of methyl mercury (micrograms per gram) in length class *l* of species *i*. Length class considerations are deemed important because of age-specific and growth-rate–specific increases in contaminant loads (Downs et al. 1998; Joiris et al. 1995). Thus, faster-growing species (higher growth coefficient *K*) have lower concentrations of methyl mercury than do slower-growing species (lower *K*), given the same size and environmental conditions.

#### Predicting methyl mercury concentrations in fish.

Methyl mercury concentrations in species are known to increase with TL. However, species at similar TLs may have different life histories and growth patterns, influencing methyl mercury concentrations. We derived a predictive relationship for fish species through a multiple regression analysis using TL, *Q*/*P* (both derived from the model), and *K* (Froese and Pauly 2004).

## Results

### Baseline simulation.

Between *t*_0_ and *t*_100_, methyl mercury concentrations in all groups increased at a declining rate, whereas concentrations in the environment increased at an average rate of 0.004 g/km^2^ per year. Predicted concentrations at *t*_100_ for most species/groups fell within the ranges reported in the literature (Table 2). However, six species/groups (four pooled groups and two individual species, herring and Greenland halibut) differed substantially (± 50% or more) between model output and literature averages. By the very nature of pooling several species into a group, groups often appear to be inadequately represented in the underlying ecosystem input data, whereas herring is poorly represented in the literature and Greenland halibut may represent a unique case.

### Impact simulations.

#### Changing fishing mortality.

**Figure 1:**
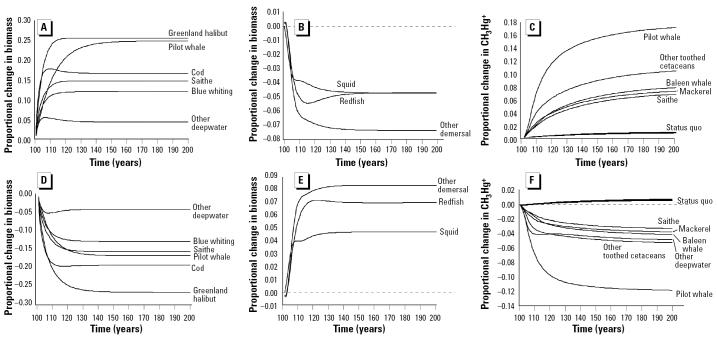
(*A–C*) Simulated decreases of 20% in fishing mortality *F*: time series of selected taxa showing the proportional increases (*A*) and the proportional decreases (*B*) in biomass of main species/functional groups, and the proportional changes in monomethyl mercury (CH_3_Hg^+^) concentrations in selected taxa (*C*). (*D–F*) Simulated increases of 20% in *F*: proportional decreases (*D*) and increases (*E*) in biomass for the main taxa, and the proportional changes in methyl mercury concentrations in selected taxa (*F* ). Status quo (no change in fishing mortality rates) represents the same taxa, but the groups do not differentiate graphically.

A 20% decrease in *F* on all targeted fish groups and pilot whales led to biomass increases for pilot whales, cod, saithe, Greenland halibut, blue whiting, and other deepwater fishes by 4–25%, relative to the constant *F* (status quo) scenario at *t*_200_ (Figure 1A). Redfish, other demersal fishes, and squid declined in biomass by 4.8, 7.4, and 4.8%, respectively (Figure 1B). The remaining species/groups had an average biomass change of −0.3% (range, −1.8% to 1.9%). Reducing *F* by 20% increased the methyl mercury concentrations in other toothed cetaceans (9.4%), pilot whales (16.0%), baleen whales (6.9%), saithe (6.0%), other deepwater fishes (8.0%), and mackerel (6.4%), relative to the status quo model at *t*_200_ (Figure 1C), whereas the remaining species/groups had an average increase in methyl mercury concentration of 4.2% (range, 2.2–4.9%).

Conversely, a 20% increase in *F* on all targeted fish groups and pilot whales had the opposite effect, resulting in decreases of 4.5–27% in biomass for pilot whales, cod, saithe, Greenland halibut, blue whiting, and other deepwater fishes (Figure 1D). Small increases in biomass were recorded for redfish, other demersal fishes, and squid (Figure 1E), whereas all other species/groups had an average increase in biomass of 0.9% (range, −1.0% to 2.9%). Increasing *F* by 20% decreased the methyl mercury concentration in other toothed cetaceans (−5.9%), pilot whales (−12.4%), baleen whales (−4.7%), saithe (−3.8%), other deepwater fishes (−5.4%), and mackerel (−4.3%), relative to the status quo scenario at *t*_200_ (Figure 1F). The remaining groups had an average decrease in methyl mercury concentration of −2.4% (range, −3.2% to −0.2%). Changing *F* by other percentages gave results that were qualitatively the same but differed quantitatively.

### Climate change scenario.

Increases in water temperature resulted in average increases in methyl mercury concentrations of 1.7% (range, 1.6–1.8%) and 4.4% (range, 4.1–4.7%) by t_200_ for projected ocean warming rates of 0.4°C and 1.0°C, respectively, per century. Simulations with the combined effects of climate change and changes in fishing mortality indicated that the two effects are cumulative.

**Figure 2:**
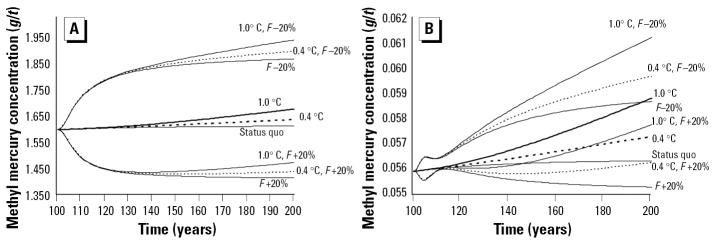
Effects of climate change and changing fishing mortality (*F*) on the methyl mercury concentrations in pilot whales (*Globicephala melas*; *A*) and cod (*Gadus morhua*; *B*). Note the significant difference in scale of the *y*-axis. Other species showed similar general trends. Increasing *F* decreased the load relative to the status quo scenario, whereas decreasing *F* had the opposite effect.

Individual species/groups responded to these changes differently, with pilot whales (Figure 2A) displaying a much greater nominal change in methyl mercury concentrations in response to changes in fishing mortality rates and climate, compared with cod (Figure 2B). However, compared with cod, the trajectory for pilot whales was dampened by the ability of pilot whales to demethylate methyl mercury.

### Human dietary intake of methyl mercury.

**Figure 3:**
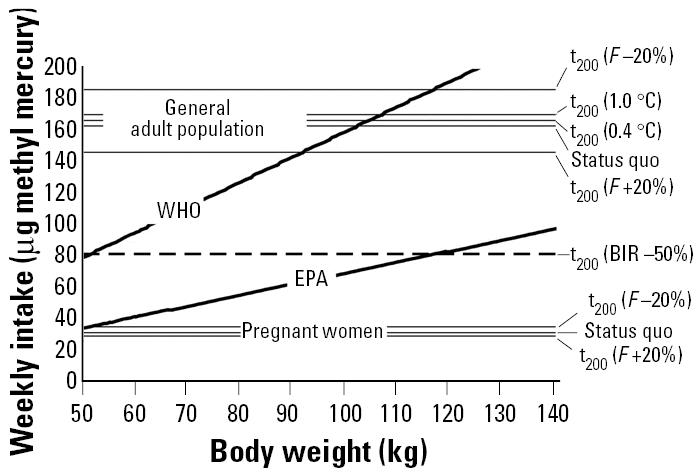
Results of the dietary analysis for the general population and pregnant women compared with the TWI limits set by the WHO (1.6 μg/kg bw) and the U.S. EPA equivalent (RfD of 0.1 μg/kg bw/day expressed as a TWI of 0.7 μg/kg bw). Under all simulations, a substantial portion of the general adult population had dietary intakes greater than the WHO limit, and all members’ consumption was greater than the limit set by the U.S. EPA. The TWI level under a simulated 50% reduction in the base inflow rate (BIR) of mercury into the environment is also shown. Pregnant women were well below the limits set by the WHO and the U.S. EPA under all scenarios. Note that the present-day scenarios for both groups at *t*_100_ are not presented because they were only marginally different than the status quo at *t*_200_.

We deemed the human dietary intake of 12 g/person/day of pilot whale meat, as observed in the 1980s (Vestergaard and Zachariassen 1987), to be the representative intake for the general population, because availability based on supply was 14.8 g/person/day in 2000 (Hagstova føroya 2001). Although consumption may have declined in recent years, no study has documented a change in dietary intake for the general population, other than for pregnant women (Weihe et al. 2003). Based on the average dietary intake of whale meat (12 g/person/day) and cod (72 g/person/day), a large portion of the general adult population exceeded the WHO limit under all simulated conditions (Figure 3). At present, individuals with a body weight of < 102 kg are above the TWI level set by the WHO for methyl mercury (FAO/WHO 2003), based on seafood consumption alone. The calculated weekly intake for the general adult population also exceeded the U.S. EPA’s limit (EPRI 2004) irrespective of body weight (Figure 3). Ocean temperature changes will increase the number of people above the WHO TWI level to individuals weighing < 105 kg and 107 kg for temperature increases of 0.4°C and 1.0°C, respectively, per century. Simulating a 20% decrease in fishing mortality resulted in members of the general population weighing < 117 kg being above the TWI set by WHO. Significantly, base environmental inflow rates of mercury would need to be reduced by approximately 50% (ignoring climate change scenarios) to ensure that levels of methyl mercury intake fall below the WHO TWI levels for current consumption patterns by the general population (Figure 3).

Interestingly, pregnant women (if consuming 1.45 g/person/day of whale meat plus 40.2 g/person/day of cod; Weihe et al. 2003) are currently, and under all simulation scenarios, well below the TWI limits set by the WHO. They are also under the limit set by the U.S. EPA, except for the very lightest individuals in cases where *F* is decreased substantially (Figure 3).

### Predictor of methyl mercury in fish.

We derived a multiple regression as


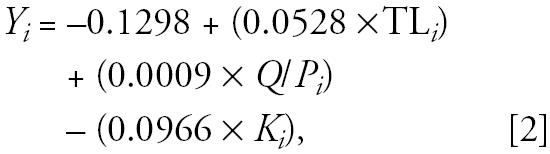


where *Y_i_* is the concentration of methyl mercury in grams per metric ton in fish species *i*; TL_i_ is the trophic level of fish species i; Q/P_i_ is the consumption to production ratio of fish species *i*; and *K_i_* is the von Bertalanffy growth parameter for fish species *i*. Although limited in its scope (df = 6), this relationship has the potential to be a good predictor of methyl mercury concentrations for fish species (*p* < 0.05; *r*^2^ = 0.905) because it incorporates relevant life history and trophic indicators, such as TL, *Q*/*P*, and *K*.

## Discussion

Methyl mercury poses substantial health risks (Fujuki 1980; UNEP 2002), and concerns of non-point-source mercury pollution was highlighted by Grandjean et al. (1992, 1997), who documented cognitive impairments in young children exposed to elevated levels of methyl mercury prenatally. This exposure was linked to the consumption of whale meat by pregnant women. Fortunately, as a result of the Grandjean et al. (1997) study, the average consumption of whale meat by pregnant women declined by approximately 90% (Weihe et al. 2003), resulting in lower levels of exposure to methyl mercury during pregnancy (Weihe et al. 2005). However, our study confirmed that by considering the average seafood diet composition as defined for the general population (Vestergaard and Zachariassen 1987), methyl mercury intakes by the nonpregnant section of the community are likely at or above the TWI levels recommend by the WHO, and substantially above the safe levels recommended by the U.S. EPA. Significantly, the analysis presented here relates to average dietary intakes and therefore does not take into account that a substantial number of people eat more than the average intake reported (i.e., are at the higher end of the intake distribution). They face substantially higher risks, especially persons with lower body weight. Hence, there is also the potential that many pregnant women exceed the U.S. EPA level, and perhaps also the WHO level. Given the present level of consumption by the general population, mercury loading of the environment would need to be reduced by approximately 50% for most of the general adult population to fall below the WHO TWI levels.

In general, simulating decreases in *F* led to an increasing trend in methyl mercury concentrations, whereas increasing *F* had the opposite effect. Although our simulations suggested that increasing fishing mortality rates would lower the concentration of methyl mercury in species/groups, it would not be sufficient to decrease methyl mercury concentrations in whale meat substantially. The dominance of methyl mercury exposure through whale meat consumption will remain a problem, irrespective of potential changes to fishing pressures. Therefore, Faroe Islanders should seriously consider reducing whale meat consumption to levels comparable with those of pregnant women (i.e., < 2 g/person/day).

Of additional concern are the likely effects of climate change, resulting in even higher concentrations of contaminants in the marine food supply of Faroe Islanders. The increasing methylation rate due to higher water temperatures will lead to continuous increases in concentrations of methyl mercury. This implies that Faroe Islanders may experience ever-increasing exposure levels, unless their dietary habits change to species with lower methyl mercury concentrations.

We have demonstrated that Ecotracer is a capable tool for tracing contaminants through all functional groups of an ecosystem, requiring relatively few toxicologic input parameters to follow the food web flow of a contaminant through all levels of an ecosystem. Significantly, our approach also lends itself to the investigation of other contaminants with significant impacts on global human health, such as dioxins and polychlorinated biphenyls (PCBs).

Although six model groups did not closely predict empirical methyl mercury concentrations, four of these entities were pooled groups (other toothed cetaceans, other deepwater fishes, benthos, and zooplankton). Pooled groups are known to be often poorly represented in the underlying input data and may be missing data for key group components. For example, other toothed cetaceans were missing methyl mercury values for killer whales (*Orcinus orca*), a species expected to have high levels of methyl mercury because of its position at the top of the food chain. In contrast, model concentrations for herring were below the value found in the literature. This may be due to the empirical data being from near-shore U.K. waters (Dixon and Jones 1994; Rowe et al. 1998) and thus not reflecting the more pelagic environment of the Faroe Islands. Greenland halibut, on the other hand, may represent a special case because the percentage of methyl mercury present in muscle tissue was reported to be between 1 and 53% (Joiris et al. 1997a), which is well below the usual 80–99% reported for most fish species (Downs et al. 1998).

Our simulations have shown that changes in methyl mercury concentrations in ecosystem components not only are due to changes in mercury input (i.e., bottom-up control) but also are influenced by top-down factors (e.g., predation and/or fishing). We also demonstrated that changes in fishing mortality can substantially alter the flow of methyl mercury in an ecosystem, by affecting the *P*/*B* ratios and the resulting trophic relations of species/groups. This might explain why tuna caught off Hawaii did not show any significant changes in methyl mercury concentrations between 1971 and 1998, despite increasing environmental loading of methyl mercury (Kraepiel et al. 2003) and growing fishing pressures (Cox et al. 2002).

Methyl mercury affects human health as a result of direct discharges and atmospheric transport. This pollutant is of particular concern to indigenous peoples of the Arctic, who often rely heavily on marine resources, and especially marine mammals, for part of their traditional diets. For example, in Greenland, approximately 43% of blood samples taken from indigenous women of reproductive age had blood mercury levels exceeding guidelines (UNEP 2003). However, increasingly pollutants found in marine resources, such as mercury, PCBs, and dioxins, are of growing concern also to westernized societies, given the growing demand for and consumption of seafood. This is illustrated by the advisory regarding seafood consumption by pregnant women issued by the U.S. Food and Drug Administration in March 2004 (U.S. FDA 2004).

Methyl mercury will continue to be of global concern as long as there are ongoing anthropogenic inputs of mercury. Our ecosystem-scale simulations suggest that substantial reductions in mercury inputs (~ 50%) would be required to ensure safe exposure levels if people such as the Faroe Islanders wish to continue their cultural dietary traditions. Unfortunately, the United States in 2002 increased the disposal or release of mercury by 10% more than the previous year (U.S. EPA 2002), whereas China’s emissions (~ 500 metric tons/year), driven primarily by coal combustion, rose by approximately 50 metric tons/year during the early 1990s (Pacyna and Pacyna 2002) and have been tracked across the Pacific Ocean to North America (UNEP 2004). Thus, anthropogenic pollution with mercury is a global problem that will continue to affect future generations in all regions of the world.

## Figures and Tables

**Table 1:** Input parameters used as starting values (*t*_0_) in the Faroe Islands Ecotracer baseline simulation (*t*_0_ to *t*_100_).

Group/species	Base inflow rate (g/km^2^/year)	Concentration (g/*t*)	Ecopath input (g/km^2^)	Direct uptake rate (g/year)	Demethylation rate (per year)
Environment	0.113	NA	3.3120	NA	NA
Other toothed cetaceans	NA	2.1150	0.0529	NA	0.20
Pilot whales	NA	1.4342	0.0737	NA	0.10
Seals	NA	1.4952	0.0105	NA	0.25
Baleen whales	NA	0.1058	0.0062	NA	0.20
Seabirds	NA	1.8985	0.0209	NA	NA
Cod	NA	0.0500	0.0285	NA	NA
Haddock	NA	0.0478	0.0345	NA	NA
Saithe	NA	0.0713	0.0435	NA	NA
Redfish	NA	0.0576	0.0613	NA	NA
Greenland halibut	NA	0.0771	0.0082	NA	NA
Other demersal fish	NA	0.0937	0.1170	NA	NA
Other deepwater fish	NA	0.2059	0.1811	NA	NA
Herring	NA	0.0246	0.0581	NA	NA
Blue whiting	NA	0.0258	0.0883	NA	NA
Mackerel	NA	0.0257	0.0138	NA	NA
Other pelagic fish	NA	0.0100	0.0765	NA	NA
Benthos	NA	0.0145	0.0281	0.0093	NA
Squid	NA	0.0173	0.1043	NA	NA
Large zooplankton	NA	0.0023	0.0377	0.0030	NA
Small zooplankton	NA	0.0009	0.0109	0.0030	NA
Phytoplankton	NA	0.0002	0.0105	0.0030	NA
Detritus	NA	0.0005	NA	NA	NA

NA, not applicable. Values were derived from the initial prebaseline simulation of the modeling routine replicating the effects of industrialization on methyl mercury concentrations in biota groups.

**Table 2:** Predicted concentrations of methyl mercury (g/metric ton) in functional groups after the 100-year simulation (*t*_0_ – *t*_100_) compared with field measurements taken from the literature.

	Literature			
Group name	Range	Mean	Model results	Literature mean vs. model results (% difference)
Other toothed cetaceans*^a^*	0.461–3.026	1.032	2.3596	128.6
Pilot whale*^b^*	1.410–1.920	1.607	1.6000	−0.4
Seals*^c^*	1.722	—	1.6684	−3.1
Baleen whales*^d^*	0.095–0.136	0.115	0.1181	3.2
Seabirds*^e^*	1.000–4.100	1.993	2.1184	6.3
Cod*^f^*	0.003–0.104	0.055	0.0558	1.5
Haddock*^g^*	0.008–0.096	0.052	0.0533	2.5
Saithe*^h^*	0.050–0.080	0.065	0.0795	22.3
Redfish*^i^*	0.024–0.148	0.072	0.0643	−10.7
Greenland halibut*^j^*	0.008–0.105	0.042	0.0860	104.8
Other demersal fish*^k^*	0.002–0.312	0.095	0.1046	10.1
Other deepwater fish*^l^*	0.056–0.336	0.139	0.2298	71.8
Herring*^m^*	0.056–0.061	0.058	0.0274	−52.8
Blue whiting	—	—	0.0288	NA
Mackerel*^n^*	0.024	0.024	0.0287	19.6
Other pelagic fish*^o^*	0.004–0.047	0.015	0.0111	−26.0
Benthos*^p^*	0.008–0.136	0.065	0.0161	−75.2
Squid*^q^*	0.008–0.024	0.016	0.0193	20.6
Large zooplankton*^r^*	0.001–0.012	0.005	0.0026	−47.7
Small zooplankton	—	—	0.0010	NA
Phytoplankton	—	—	0.0002	NA
Detritus	—	—	0.0005	NA
Environment*^s^*	3.612	3.612	3.6078	−0.4

Abbreviations: —, not reported in literature; NA, not applicable.

a Das et al. 2003; Holsbeek et al. 1999; Joiris et al. 1991; Siebert et al. 1999.

b Caurant et al. 1996; Dam and Bloch 2000.

c Das et al. 2003.

d Dietz et al. 1996; Hansen et al. 1990.

e Joiris et al. 1997c.

f Joiris et al. 1997a; Mormede and Davies 2001; Rowe et al. 1998; Zauke et al. 1999.

g Joiris et al. 1995; Rowe et al. 1998; Zauke et al. 1999.

h Julshamn and Grahl-Nielsen 1996; Zauke et al. 1999.

i Zauke et al. 1999.

j Joiris et al. 1997a; Zauke et al. 1999.

k Dixon and Jones 1994; Joiris et al. 1997a; Mormede and Davies 1998, 2001; Nixon et al. 1994; Rowe et al. 1998; Zauke et al. 1999.

l Cronin et al. 1998; Mormede and Davies 2001; Rowe et al. 1998.

m Dixon and Jones 1994; Rowe et al. 1998.

n Rowe et al. 1998.

o Joiris et al. 1995; Roméo et al. 1999.

p Canli and Furness 1995; Dietz et al. 1996; Joiris et al. 1997a; Nixon et al. 1994; Rowe et al. 1998.

q Frodello et al. 2000.

r Joiris et al. 1997b; Ritterhoff and Zauke 1997.

s Mason R, personal communication.
